# Q Fever Endocarditis and a New Genotype of *Coxiella burnetii*, Greece

**DOI:** 10.3201/eid2610.191616

**Published:** 2020-10

**Authors:** Ioulia Karageorgou, Nektarios Kogerakis, Stavroula Labropoulou, Sophia Hatzianastasiou, Andreas Mentis, George Stavridis, Emmanouil Angelakis

**Affiliations:** Hellenic Pasteur Institute, Athens, Greece (I. Karageorgou, S. Labropoulou, A. Mentis, E. Angelakis);; Onassis Cardiac Surgery Center, Athens (N. Kogerakis, S. Hatzianastasiou, G. Stavridis);; Aix Marseille Université, Marseille, France (E. Angelakis)

**Keywords:** Q fever, endocarditis, acute Q fever endocarditis, Coxiella burnetii, bacteria, genotype, multispacer sequence typing, MST, Greece

## Abstract

Underdiagnosis of *Coxiella burnetii* infections in Greece is possible because of lack of awareness by physicians, and most suspected cases are in patients with no bovine contact. We found serologic evidence of *C. burnetii* infection throughout Greece and identified a new *C. burnetii* genotype in the aortic valve of a patient with Q fever endocarditis.

Q fever is a worldwide zoonosis caused by an obligate intracellular bacterium, *Coxiella burnetii* (*1*,*2*). Although the classification of *C. burnetii* by the Centers for Disease Control and Prevention (Atlanta, GA, USA) as a potential bioterrorism agent resulted in the disease becoming reportable in many countries (*3*), Q fever is not considered a public health problem in Greece, and few cases have been recorded (*3*).

Our referent laboratory for the diagnosis of Q fever was deployed in the Hellenic Pasteur Institute in February 2019. We tested serum samples from all patients by using an immunofluorescence assay (IFA) for *C. burnetii* phase I and II antigens as described (*4*,*5*). Patients are classified as having acute Q fever; persistent, focalized *C. burnetii* infection; or evidence of past infection (*6*). Moreover, anticardiolipin IgG is routinely measured for patients given a diagnosis of acute Q fever (*6*).

During the first 7 months of testing, we received 209 serum samples from patients suspected of having Q fever. We provided diagnoses of acute Q fever for 1 (0.5%) patient and persistent *C. burnetii* focalized endocarditis for 2 (1.0%) patients; 12 (6.0%) patients showed evidence of *C. burnetii* infection. The patient given a diagnosis of acute Q fever also had high levels of anticardiolipin IgG (>140 GPLU). Further investigation also showed large, transient, aortic vegetation. Thus, this patient was considered as possibly having acute Q fever endocarditis (*4*,*7*), but contact with the patient was lost.

Epidemiologic information was obtained for 102 patients, including all patients with a positive IFA result for *C. burnettii*. This information showed that only 22% of these patients reported previous contact with bovids. Most patients reported a previous tick bite (35%); contact with cats (16%), dogs (7%), rats (4%), or other animals (7%). In addition, 9% of these patients reported no animal contact.

We provide a detailed history for 1 patient given a diagnosis of persistent *C. burnetii* focalized endocarditis. A 45-year-old sheepherder, a resident of a rural area in southern Greece, came to the local district hospital with a 2-week history of spiking fevers, peripheral edema, and night sweats. He reported nonspecific symptoms gradually leading to anorexia and debilitating weakness for the previous year.

Cardiac ultrasound showed a severely regurgitant bicuspid aortic valve, a paravalvular abscess (2.6 cm × 1.6 cm), aortic root dilatation (5.3 cm), and vegetations. Cardiac computed tomography confirmed the ultrasound findings. IFA results were positive for *C. burnetii*: phase I IgG titer 1:3,200, phase I IgM titer 0; and phase II IgG titer 1:3,200, phase II IgM titer 0. This serum sample was negative for *C. burnetii* by real-time PCR for insertion sequence (IS) 1111 and the IS30A spacers (*8*). Thus, we provided a diagnosis of *C. burnetii* endocarditis, and the patient was transferred to a tertiary care center for surgical management.

The patient underwent an aortic root replacement (Bentall procedure) with pericardial composite graft after extensive debridement and reconstruction of the root with the use of autologous pericardium. His aortic valve was positive for *C. burnetii* for IS1111 and IS30A spacers by real-time PCR. Multispacer sequence typing (MST) was performed as described and consisted of 10 different spacers of the *C. burnetii* genome: Cox2, 5, 6, 18, 20, 22, 37, 51, 56, and 57 (*5*). We identified a new MST genotype (MST65) by using web-based MST database (http://ifr48.timone.univ-mrs.fr/MST_Coxiella/mst) (Figure, panel A).

**Figure:**
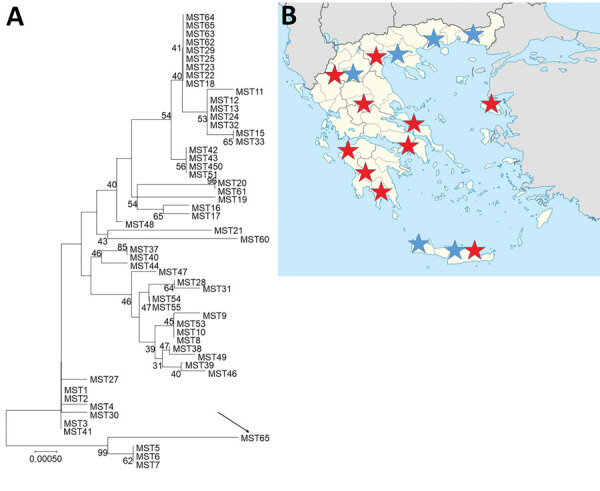
Investigation of Q fever endocarditis, Greece. A) Neighbor-joining tree of *Coxiella burnetii* genotypes determined by multispacer sequence typing. Analysis was performed by using MEGA version 7 software (https://www.megasoftware.net) and the neighbor-joining method (maximum composite likelihood method) with 1,000 replicates. Numbers along branches are bootstrap values. Arrow indicates new genotype from Greece. Scale bar indicates nucleotide substitutions per site. B) Seroepidemiologic evidence of *C. burnetii* cases in Greece. Blue stars indicate previous studies, and red stars indicate this study. MST, mutispacer type.

The patient was given oral doxycycline (100 mg 2×/d) and hydroxychloroquine (200 mg 3×/d) for >24 months (*9*). A convalescence-phase serum sample obtained after 6 months of treatment was positive for *C. burnetii*: phase I IgG titer 1:800, phase I IgM titer 0, and phase II IgG titer 1:800, phase II IgM 0.

Our preliminary data show that physicians in Greece are not familiar with Q fever because most of the suspected cases were in patients without bovine contact. A limitation of our study was that culture was not performed because of the absence of a Biosafety Level 3 laboratory. The fact that we did not provide diagnoses of classic, acute Q fever showed that *C. burnetii* infection is suspected mostly in culture-negative serious endocarditis case-patients. Moreover, we identified a new *C. burnetii* genotype in the aortic valve of a patient who had Q fever endocarditis. Recently, it was found that *C. burnetii* genotype 32 is circulating in sheep and goat in 8 different areas of Greece (*10*). The clinical manifestations of Q fever depend, at least in part, on the *C. burnetii* genotype (*5*). However, although acute clinical manifestations are strain-specific, all genotypes have been associated with endocarditis (*5*).

We raise the question of underdiagnosis of C. *burnetii* infections in Greece. Our data have affected local clinical practice because we found serologic evidence of C. *burnetii* infection throughout most of Greece (Figure, panel B).
